# Bioinformatics analysis of the association between obesity and gastric cancer

**DOI:** 10.3389/fgene.2024.1385559

**Published:** 2024-07-01

**Authors:** Xiaole Ma, Miao Cui, Yuntong Guo

**Affiliations:** ^1^ Department of Gastrointestinal Surgery, First Hospital of Shanxi Medical University, Taiyuan, China; ^2^ Department of Geriatrics, First Hospital of Shanxi Medical University, Taiyuan, China

**Keywords:** gastric cancer, obesity, bioinformatics, differentially expressed genes, hub genes

## Abstract

**Background:**

Obesity and gastric cancer (GC) are prevalent diseases worldwide. In particular, the number of patients with obesity is increasing annually, while the incidence and mortality rates of GC are ranked high. Consequently, these conditions seriously affect the quality of life of individuals. While evidence suggests a strong association between these two conditions, the underlying mechanisms of this comorbidity remain unclear.

**Methods:**

We obtained the gene expression profiles of GSE94752 and GSE54129 from the Gene Expression Omnibus database. To investigate the associated biological processes, pathway enrichment analyses were conducted using Gene Ontology and Kyoto Encyclopedia of Genes and Genomes for the shared differentially expressed genes in obesity and GC. A protein–protein interaction (PPI) network was subsequently established based on the Search Tool for the Retrieval of Interacting Genes (STRING) database, followed by the screening of the core modules and central genes in this network using Cytoscape plug-in MCODE. Furthermore, we scrutinized the co-expression network and the interplay network of transcription factors (TFs), miRNAs, and mRNAs linked to these central genes. Finally, we conducted further analyses using different datasets to validate the significance of the hub genes.

**Results:**

A total of 246 shared differentially expressed genes (209 upregulated and 37 downregulated) were selected for ensuing analyses. Functional analysis emphasized the pivotal role of inflammation and immune-associated pathways in these two diseases. Using the Cytoscape plug-in CytoHubba, nine hub genes were identified, namely, *CXCR4*, *CXCL8*, *CXCL10*, *IL6*, *TNF*, *CCL4*, *CXCL2*, *CD4*, and *CCL2*. *IL6* and *CCL4* were confirmed as the final hub genes through validation using different datasets. The TF-miRNA-mRNA regulatory network showed that the TFs primarily associated with the hub genes included RELA and NFKB1, while the predominantly associated miRNAs included has-miR-195-5p and has-miR-106a-5p.

**Conclusion:**

Using bioinformatics methods, we identified two hub genes from the Gene Expression Omnibus datasets for obesity and GC. In addition, we constructed a network of hub genes, TFs, and miRNAs, and identified the major related TFs and miRNAs. These factors may be involved in the common molecular mechanisms of obesity and GC.

## Introduction

Obesity has become a serious global public health problem that primarily affects adults, though childhood obesity cannot be ignored. The prevalence of obesity is increasing yearly worldwide and has almost tripled since 1975. In 2016, >1.9 billion adults were overweight, while >650 million individuals were obese (World Health Organization, 2021). The number of deaths due to overweight and obesity is also increasing. In 2017, >4 million individuals expired due to overweight or obesity (World Health Organization, 2024). Obesity is strongly associated with several chronic diseases, including hypertension, type 2 diabetes, cardiovascular disease, dyslipidemia, and osteoarthritis (Pi-Sunyer, 2009). Additionally, obesity has been implicated in the genesis of numerous cancer types, including colorectal, gastric cardia, liver, gallbladder, pancreatic, renal, and esophageal adenocarcinoma (da Cruz et al., 2022; Kolb et al., 2016; Madka et al., 2023). Gastric cancer (GC) is the fifth most common type of cancer worldwide and the fourth leading cause of cancer-related death (Sung et al., 2021). Some observational studies have reported a positive association between obesity and gastric cardia cancer (Chen et al., 2013; Turati et al., 2013). Other studies have reported a positive association between obesity and non-cardia gastric cancer (Mao et al., 2017). Xing A et al. conducted a large-scale, two-sample Mendelian randomization analysis in order to confirm the causal relationship between obesity and gastric cancer (Xing et al., 2023). However, the specific biological mechanisms underlying this association remain unclear.

A number of studies have demonstrated the important role of inflammation in the development of obesity and cancer (Iyengar et al., 2016). The tumor microenvironment (TME) shares similar features with a healing wound (Mantovani et al., 2008). Similar to the wound healing process following tissue injury, inflammation in adipose tissue creates an environment that promotes tumor formation (de Visser et al., 2006). In addition to inflammatory cells, cancer cells can be involved in the tissue repair process, thus promoting tumor growth and infiltration. Obesity is widely recognized as a contributing factor to chronic inflammation, both systemically and within tissues (Iyengar et al., 2015). In obese individuals, many immune cells (e.g., macrophages and lymphocytes) are present in adipose tissues (Connaughton et al., 2016). Consequently, the adipose tissue in obese individuals resembles chronically injured tissue and has the capacity to release pro-inflammatory substances, potentially facilitating tumor growth.

In this study, a bioinformatics approach was utilized to screen for shared differentially expressed genes (DEGs) in patients with obesity and GC using Gene Expression Omnibus (GEO) datasets. A total of 209 upregulated genes and 37 downregulated genes were shared between the two diseases. Enrichment analysis, including Gene Ontology (GO) and Kyoto Encyclopedia of Genes and Genomes (KEGG), revealed a predominant enrichment of these genes in inflammatory and immune-related pathways. Through the construction of a protein–protein interaction (PPI) network and Cytoscape module analysis, nine hub genes were identified using six algorithms from the CytoHubba plugin. Subsequently, miRNA-gene-transcription factor (miRNA-gene-TF) regulatory networks were constructed for these nine hub genes. Finally, we validated the nine hub genes using different datasets. We sought to explore common molecular markers between obesity and GC.

## Materials and methods

### Raw data collection

We conducted a search for gene expression datasets related to GC and obesity using keywords in the GEO database (http://www.ncbi.nlm.nih.gov/geo) (Edgar et al., 2002). The GEO was created and is maintained by the National Center for Biotechnology Information (Bethesda, MD, United States of America). It contains high-throughput gene expression data and microarray data submitted by research organizations worldwide. The purpose of this search was to identify relevant datasets that could provide valuable insights into gene expression patterns associated with GC and obesity. Based on the inclusion criteria we specified, two microarray datasets were downloaded: GSE54129 and GSE94752. These datasets were chosen because they were widely used in previous literature (Duarte et al., 2023; Yi et al., 2023). Both datasets were based on different Affymetrix platforms: GPL570 and GPL11532. The GSE54129 dataset comprises 111 patients with GC who underwent subtotal gastrectomy, along with 21 volunteers who underwent gastroscopy for health examination. This dataset provides gene expression profiles specifically related to GC. The GSE94752 dataset includes nine lean patients and 39 patients with obesity. This dataset focuses on the comparison of gene expression patterns between lean and obese individuals, providing insights into the molecular mechanisms associated with obesity. By analyzing these two datasets, we can gain valuable information regarding gene expression changes in GC and the molecular differences between lean and obese individuals.

### Identification of DEGs

The Limma package (version: 3.58.1) within R software was employed to examine the differential expression of mRNAs. We set “*p* < 0.05 and fold-change ≥0.5” as the cut-off criteria for the identification of differentially expressed mRNAs. Genes with more than one probe set were removed. The Venn package (version: 1.7.3) of R software was used to obtain overlapping DEGs between the two datasets, which were subsequently analyzed.

### Enrichment analyses of DEGs

The DEGs were subjected to enrichment analyses to evaluate their roles and interactions within biological pathways. Using the R package, we analyzed Gene Ontology (GO) and Kyoto Encyclopedia of Genes and Genomes (KEGG) pathways for upregulated and downregulated DEGs, respectively. Adjusted *p*-values <0.05 indicate statistically significant differences. The *p*-values are adjusted by Benjamini and Hochberg.

### Protein–protein interaction (PPI) network construction and module analysis

The Search Tool for the Retrieval of Interacting Genes (STRING) database (version: 12.0) (Franceschini et al., 2013) is a valuable resource for investigating PPIs and constructing PPI networks. The STRING database utilizes information from multiple public databases to generate a comprehensive network of protein interactions and allows its visualization. Interactions with a cumulative score >0.9 were deemed statistically significant. The PPI network data were imported into Cytoscape (http://www.cytoscape.org) (version 3.9.1) (Smoot et al., 2011) for further analysis. Screening of key functional modules was performed using the Molecular Complex Detection (MCODE) plugin in Cytoscape. We established the selection parameters as follows: K-core = 2, degree cutoff = 2, maximum depth = 100, and node score cutoff = 0.2. Subsequently, we conducted KEGG and GO analyses for the genes implicated in these modules.

### Identification and evaluation of hub genes

Hub genes were screened using the CytoHubba plugin in Cytoscape. We used six commonly used algorithms (Maximal Clique Centrality, Maximum Neighborhood Component, Degree, Closeness, Radiality, EcCentricity). The genes obtained from these algorithms were subsequently intersected using the online Venn tool (https://bioinfogp.cnb.csic.es/tools/v-enny/index.html) (Bardou et al., 2014) to identify common hub genes. Thereafter, these hub genes were imported into the GeneMANIA tool (http://www.genemania.org/) (Warde-Farley et al., 2010) to construct the co-expression network; the GeneMANIA tool is a trusted instrument for the construction of co-expression networks and the identification of intrinsic relationships within gene clusters. It utilizes genomics and proteomics data to identify functionally similar genes, and predicts the function of these genes.

### Analysis of the regulatory network involving transcription factors (TFs), miRNAs, and mRNAs

We sought to understand the interactions between the acquired hub genes. To this end, we utilized the TRRUST (transcriptional regulatory relationships unraveled by sentence-based text-mining) (version:2) database to obtain TF-target interactions. The TRRUST database utilizes text mining techniques to extract information from scientific literature, and curates a comprehensive collection of experimentally validated TF-target interactions (Han et al., 2018). In addition, we utilized miRWalk (a public miRNA target gene database including information of multiple species) (version: 3), which provides a comprehensive collection of experimentally validated and predicted miRNA-target interactions (Sticht et al., 2018). In this analysis, we considered only predicted miRNA-target interactions that have been verified by experiments to improve the accuracy of the results. The interactions between these two targets were subsequently fused using Cytoscape to construct a comprehensive TF-miRNA-mRNA regulatory network.

### Validation of hub genes

To validate the reliability of our findings, we performed the Wilcoxon test to assess the expression of hub genes. For this purpose, we utilized the GSE25401 dataset, which comprises 26 nonobese samples and 30 obese samples. Additionally, we employed the GSE220917 dataset, which includes 18 GC samples and five normal samples, for further verification. *p*-values <0.05 indicate statistically significant differences.

## Results

### Detection of DEGs

**FIGURE 1:**
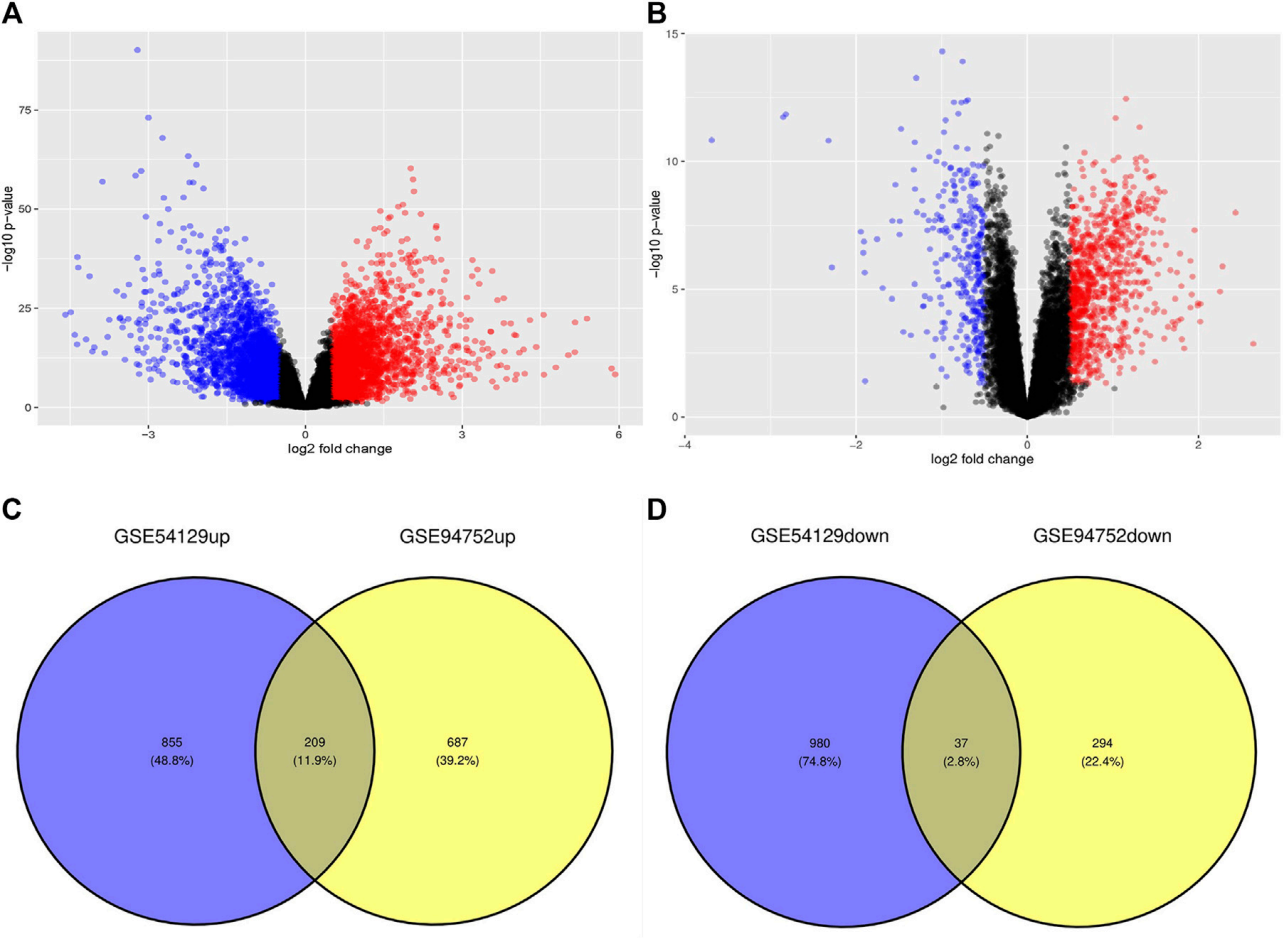
Volcano diagram and Venn diagram. **(A)** The volcano map of GSE54129. **(B)** The volcano map of GSE94752. Upregulated genes are marked in light red; downregulated genes are marked in light blue. **(C, D)** The two datasets showed an overlap of 246 DEGs, including 209 upregulated genes and 37 downregulated genes.

We identified 2,081 and 1,227 DEGs in the GSE54129 and GSE94752 GEO datasets, respectively (Figures 1A, B). A Venn diagram computation revealed 209 overlapping upregulated genes and 37 overlapping downregulated genes in these datasets (Figures 1C, D).

### Functional characteristic analysis of shared DEGs

**FIGURE 2:**
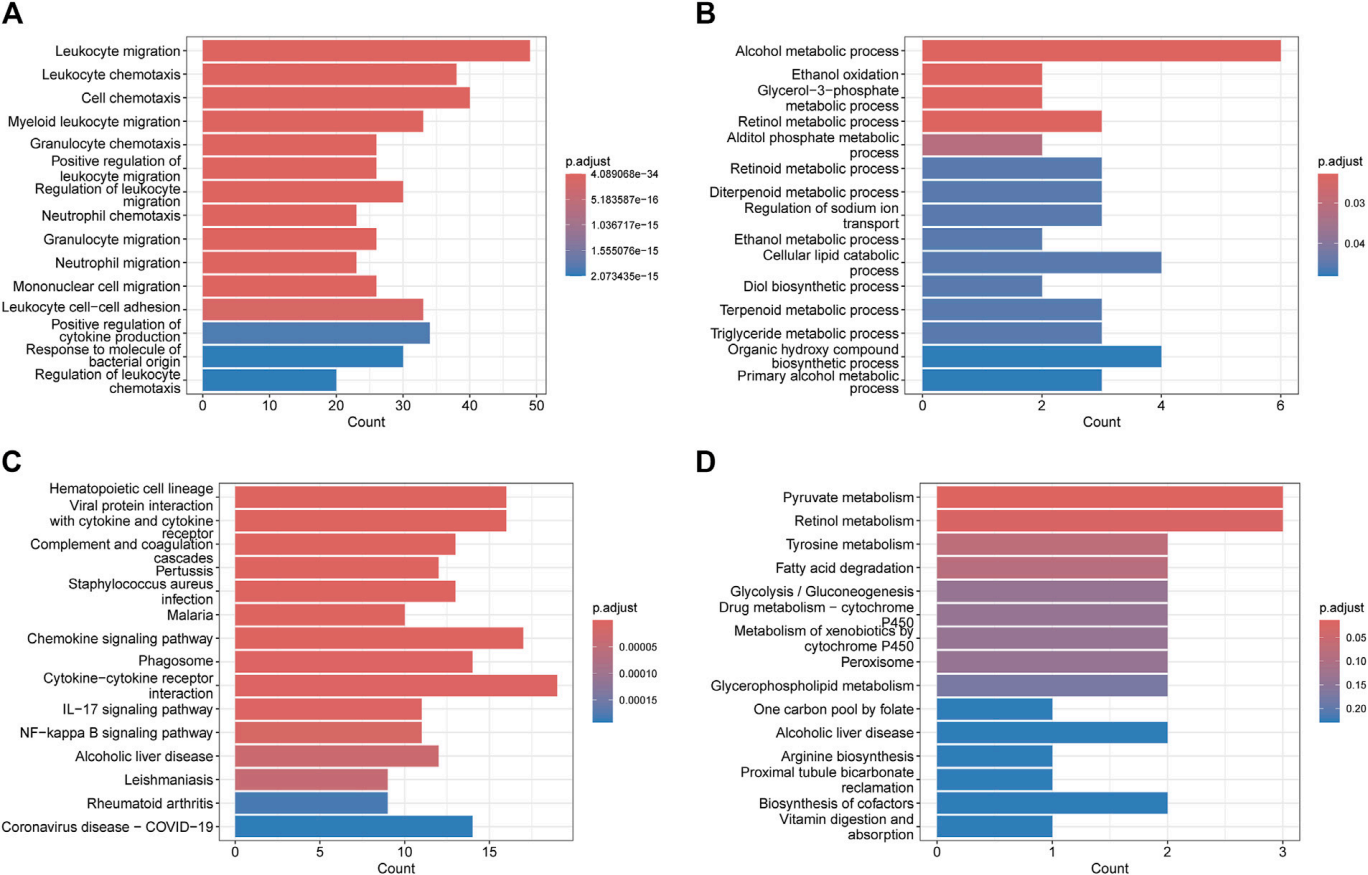
Functional enrichment: **(A)** enrichment result of upregulated DEGS GO term. **(B)** enrichment result of downregulated DEGS GO term. **(C)** enrichment result of upregulated DEGS KEGG pathway. **(D)** enrichment result of downregulated DEGS KEGG pathway.

The GO analysis revealed that the upregulated genes were predominantly enriched in processes related to Leukocyte migration (p. adjust = 4.09E-34), Leukocyte chemotaxis (p. adjust = 8.56E-30), Cell chemotaxis (p. adjust = 4.59E-28), and Myeloid leukocyte migration (p. adjust = 1.02E-23) (Figure 2A). The downregulated genes were mainly enriched in Alcohol metabolic process (p. adjust = 0.02), Ethanol oxidation (p. adjust = 0.02), Glycerol-3-phosphate metabolic process (p. adjust = 0.02), and Retinol metabolic process (p. adjust = 0.02) (Figure 2B). Based on the KEGG pathway analysis, the upregulated genes were primarily enriched in the Hematopoietic cell lineage (p. adjust = 2.14E-10), Viral protein interaction with cytokine and cytokine receptor (p. adjust = 2.14E-10), Chemokine signaling pathway (p. adjust = 1.70E-07), and Cytokine-cytokine receptor interaction (p. adjust = 3.22E-06) (Figure 2C). The downregulated genes were predominantly enriched in the Pyruvate metabolism (p. adjust = 0.01), Retinol metabolism (p. adjust = 0.02) (Figure 2D).

### Construction of a PPI network and module analysis

**FIGURE 3:**
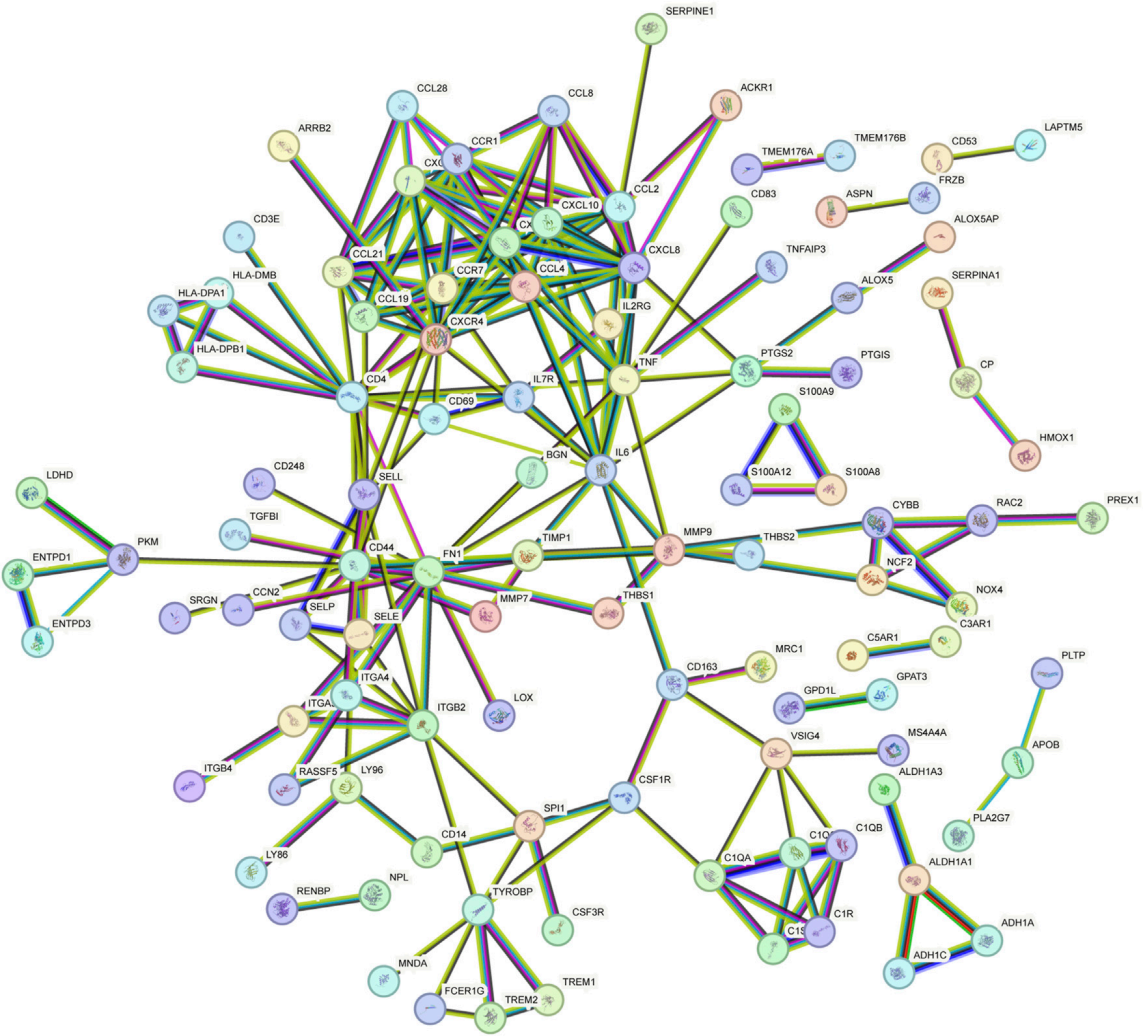
PPI network constructed using the STRING database.

**FIGURE 4:**
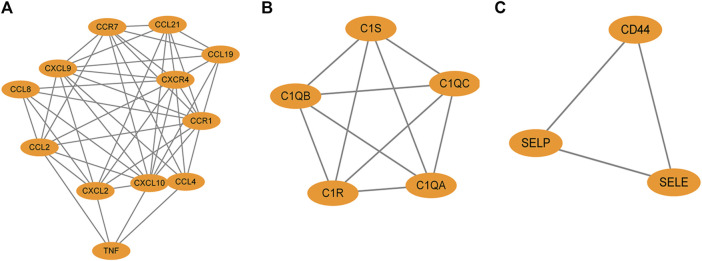
The protein interaction network obtained by analyzing PPI with Cytoscape plugin MCODE, **(A–C)** represent the three sub-modules obtained by MCODE plugin.

**FIGURE 5:**
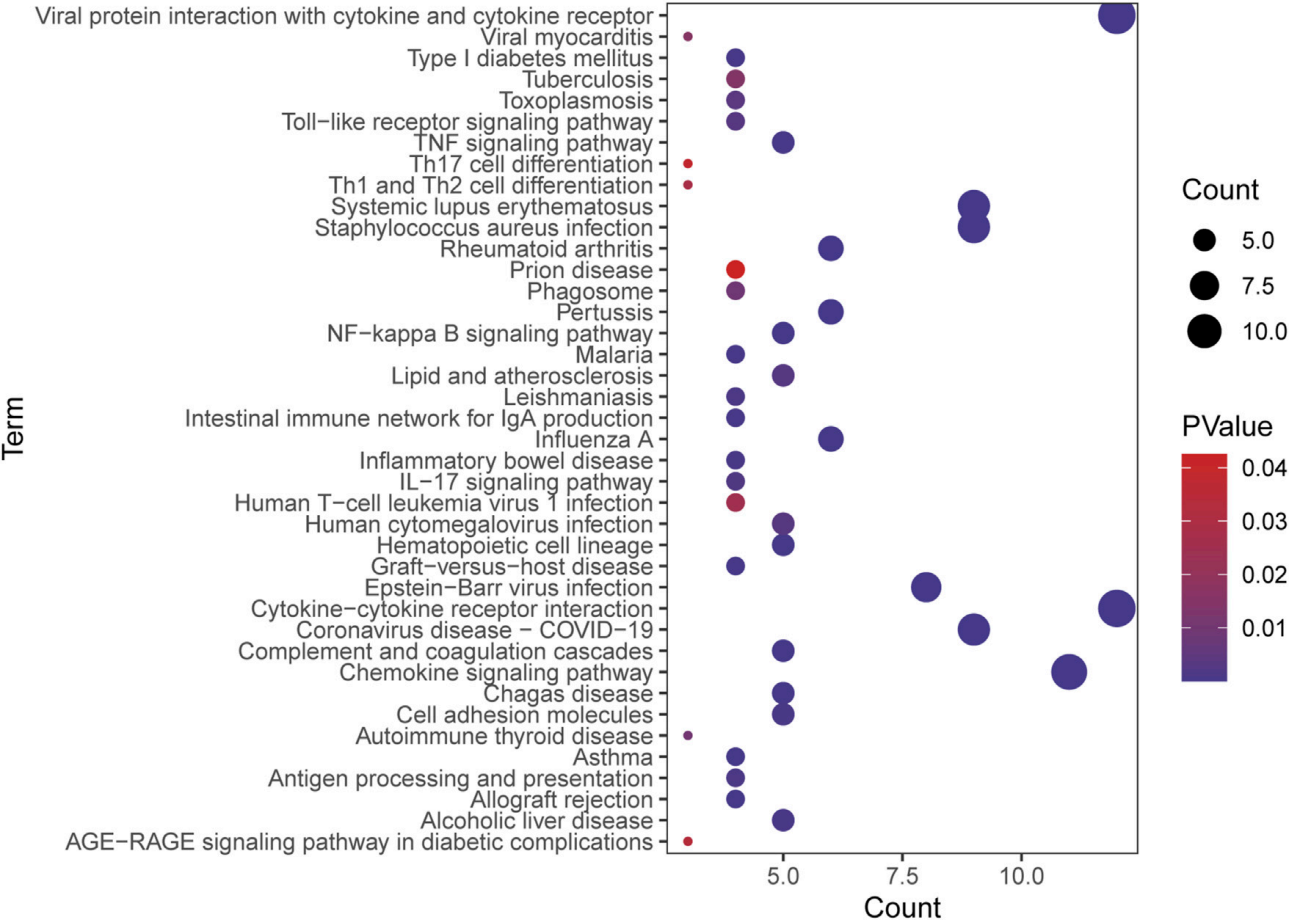
Bubble plot of KEGG enrichment analysis results.

**FIGURE 6:**
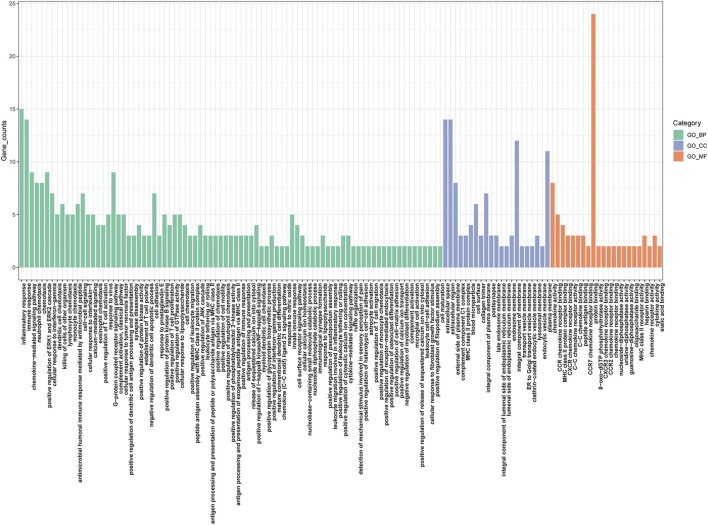
Bar plot of GO enrichment analysis results. MF, Molecular Function; BP, Biological Process; CC, Cellular Component.

The PPI network of overlapping DEGs comprised 245 nodes and 213 interaction pairs (Figure 3). Three highly interconnected gene modules were identified, which included 20 common DEGs and 59 interaction pairs (Figure 4). The KEGG analysis revealed that these modules were predominantly involved in cytokine-cytokine receptor interaction, viral protein interaction with cytokine and cytokine receptor, and the chemokine signaling pathway (Figure 5). The GO analysis demonstrated that these genes are associated with various biological processes, cellular components, and molecular functions. Specifically, they are involved in the inflammatory response, immune response, and chemokine-mediated signaling pathway (biological processes), and are associated with cell maturation, extracellular space, and the extracellular region (cellular components). Furthermore, they exhibit protein binding, plasma membrane association, and chemokine activity (molecular functions) (Figure 6).

### Identification and analysis of hub genes

**FIGURE 7:**
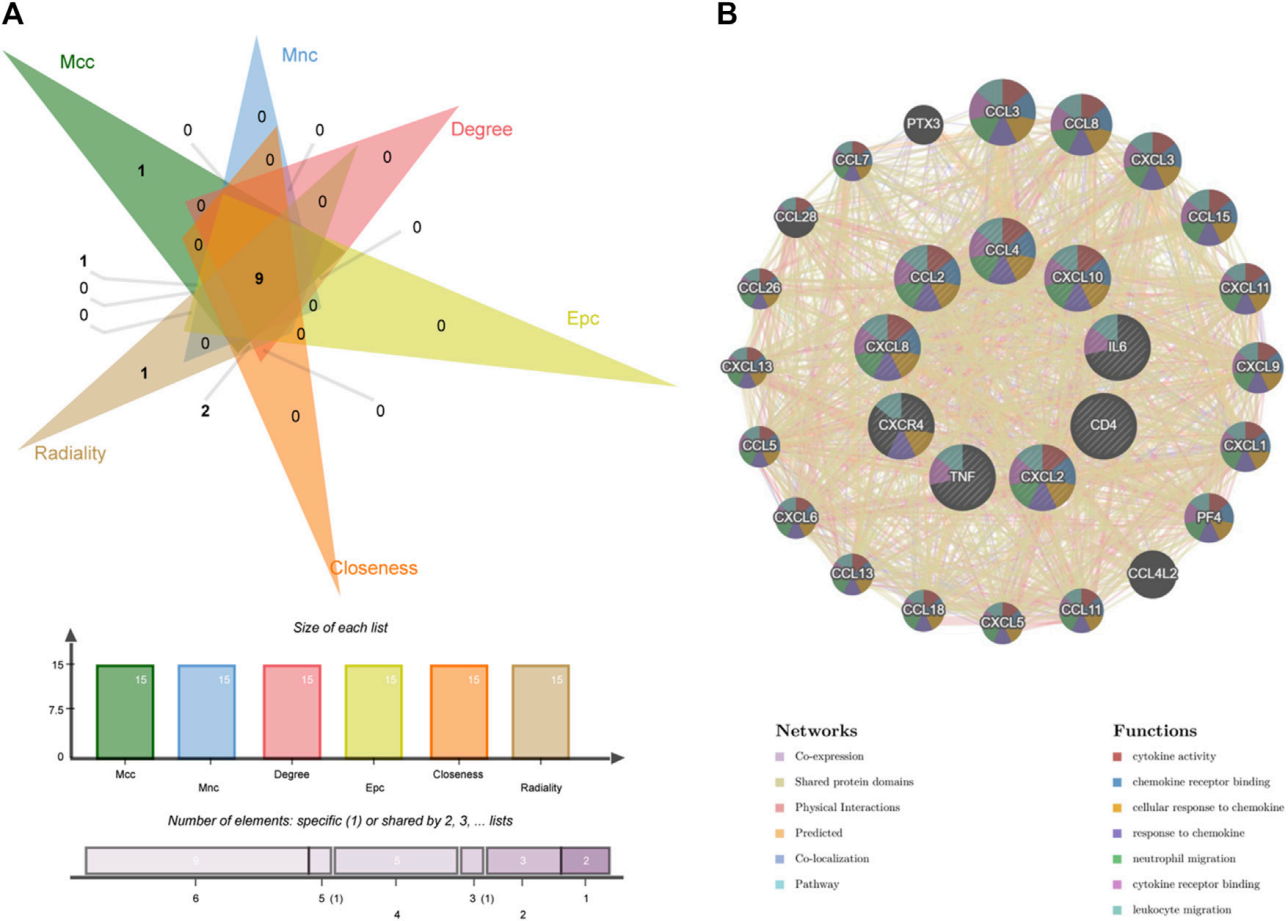
Venn diagram and co-expression network of hub genes. **(A)** The Venn diagram showed that six algorithms have screened out nine overlapping hub genes. **(B)** Hub genes and their co-expression genes were analyzed via GeneMANIA.

We identified the top 15 key genes, which are shown in Table 1. Using the Venn tool online, we obtained nine overlapping hub genes, namely, *CXCR4*, *CXCL8*, *CXCL10*, *IL6*, *TNF*, *CCL4*, *CXCL2*, *CD4*, and *CCL2* (Figure 7A). Table 2 shows the full names and functions of these genes. Using the GeneMANIA database, we explored genes with similar functions to those genes, established the co-expression networks, and analyzed their major biological pathways (Figure 7B). The PPI network included physical interactions (11.7%), co-expression (59.03%), predicted (6.08%), co-localization (5.91%), and pathway (0.99%). This network showed that the nine hub genes had significant interactions with *CCL3*, *CCL8*, *CXCL3*, *CXCL15*, *CXCL11*, and other important genes. The biological functions of these genes are related to the regulation of inflammatory response, cytokine binding, and leukocyte migration.

**TABLE 1:** The top 15 hub genes identified using CytoHubba.

MCC	MNC	Degree	Closeness	Radiality	EcCentricity
*CXCL10*	*CXCL10*	*CXCR4*	*IL6*	*IL6*	*IL6*
*CXCR4*	*CXCR4*	*CXCL10*	*CD4*	*FN1*	*FN1*
*CCR1*	*CXCL8*	*CXCL8*	*FN1*	*CD4*	*CD4*
*CXCL8*	*CCR7*	*IL6*	*CD44*	*TNF*	*TNF*
*CXCL9*	*IL6*	*CD4*	*TNF*	*CD44*	*CXCR4*
*CCR7*	*CCR1*	*FN1*	*CXCR4*	*CXCR4*	*SELL*
*CCL2*	*CXCL9*	*CCR7*	*CXCL8*	*SELL*	*TIMP1*
*CXCL2*	*CCL2*	*TNF*	*CXCL10*	*TIMP1*	*MMP9*
*CCL19*	*CXCL2*	*CD44*	*SELL*	*MMP9*	*CXCL8*
*CCL21*	*TNF*	*CCL2*	*CCR7*	*CXCL8*	*CXCL10*
*CCL4*	*CD4*	*CCR1*	*CCL2*	*CXCL10*	*CCL2*
*CCL8*	*CD44*	*CXCL9*	*CXCL2*	*CCL2*	*CCL4*
*IL6*	*CCL21*	*CXCL2*	*MMP9*	*CCL4*	*CXCL2*
*TNF*	*CCL4*	*CCL21*	*CCL4*	*ITGB2*	*ITGA4*
*CD4*	*FN1*	*CCL4*	*ITGB2*	*CXCL2*	*CD163*

MCC, maximal clique centrality; MNC, maximum neighborhood component.

**TABLE 2:** Details of the hub genes.

No.	Gene symbol	Full name	Function
1	*CXCR4*	C-X-C motif chemokine receptor 4	The gene encodes a distinct receptor, *CXCR4*, which specifically binds to the chemokine stromal cell-derived factor-1. *CXCR4* is a G-protein-coupled receptor (*GPCR*) composed of 352 amino acids and characterized by a seven-transmembrane domain structure. It exhibits widespread expression in numerous tissues and organs
2	*CXCL8*	C-X-C motif chemokine ligand 8	This gene belongs to the CXC-type chemokine family and plays a crucial role as a key mediator in the inflammatory response. It can be secreted by various cell types, including leukocytes, fibroblasts, endothelial cells, and malignant tumor cells. Its biological effects are mediated through the binding of two *GPCRs*, namely, *CXCR1* and *CXCR2*
3	*CXCL10*	C-X-C motif chemokine ligand 10	*CXCL10* is a member of the *CXC* chemokine family. It is produced and secreted by several cell types in the body, including monocytes, endothelial cells, and fibroblasts. *CXCL10* serves multiple functions, including the chemotaxis of monocytes/macrophages, natural killer cells, and dendritic cells. Additionally, it inhibits bone marrow colony formation and angiogenesis (the process of new blood vessel formation)
4	*IL6*	Interleukin 6	*IL6* is a cytokine closely associated with inflammation. It plays a crucial role in regulating immune and inflammatory responses within the body. *IL6* can be produced by various lymphocytes, including T-lymphocytes, B-lymphocytes, and others. It exerts its effects by promoting the proliferation and differentiation of various cell types
5	*TNF*	Tumor necrosis factor	This gene is a prototypical pro-inflammatory cytokine that belongs to the *TNF* superfamily. It is primarily produced by macrophages in the body. It plays a crucial role in regulating diverse biological processes, such as cell proliferation, differentiation, and apoptosis
6	*CCL4*	C-C motif chemokine ligand 4	This gene encodes a CC chemokine that specifically binds to the C-C motif chemokine receptor 5 (*CCR5*). It is considered one of the key human immunodeficiency virus suppressors produced by CD8^+^ T cells. It functions by attracting and guiding various immune cells, including natural killer cells and monocytes, through the process of chemotaxis
7	*CXCL2*	C-X-C motif chemokine ligand 2	*CXCL2*, also known as macrophage inflammatory protein-2 (*MIP-2*), is a chemokine that plays multiple roles in the cellular and immune systems. It functions by inducing cell chemotaxis, promoting the release of inflammatory mediators, modulating the immune response, and facilitating tissue repair
8	*CD4*	CD4 molecule	*CD4* is a crucial gene that encodes a membrane glycoprotein with diverse functions in both *in vivo* and *in vitro* immune responses. It is primarily expressed on the surface of T helper cells, regulatory T cells, monocytes, macrophages, and dendritic cells. *CD4* plays a pivotal role in promoting T cell activation and development, thereby contributing to the overall immune response
9	*CCL2*	C-C motif chemokine ligand 2	*CCL2*, a chemokine belonging to the CC subfamily, plays a crucial role in chemotaxis of monocytes, macrophages, and T-lymphocytes. By orchestrating these migratory processes, *CCL2* contributes to the immune system’s defense against invading microorganisms. Additionally, *CCL2* exerts its effects across various cellular functions, thereby resulting in a wide range of physiological impacts. Its expression is observed in multiple locations throughout the body, further highlighting its significance in immune responses

### TF-miRNA-mRNA regulatory network analysis

**FIGURE 8:**
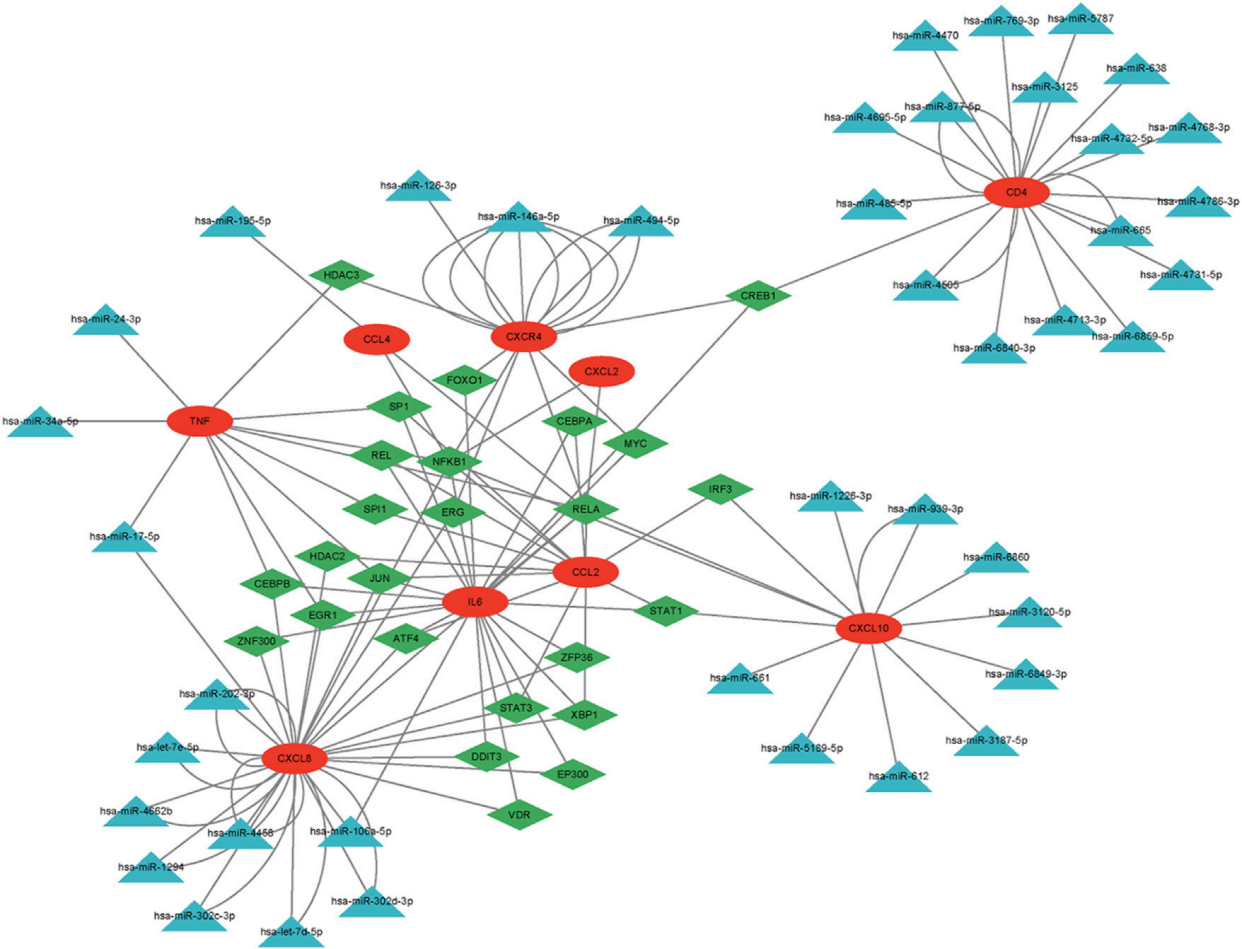
The regulatory network of nine hub genes. Red circles represent hub genes; green diamonds represent transcription factors (TFs); blue triangles represent microRNAs (miRNAs).

Using the TRRUST and miRWalk databases, we identified 25 TFs and 67 miRNAs that potentially regulate the expression of hub genes. To investigate the underlying regulatory mechanism, we constructed a regulatory network that integrates these genes. Furthermore, we utilized information on miRNA, TF, and mRNA interactions to create an “TF-miRNA-mRNA” regulatory network, consisting of 67 miRNAs, nine mRNAs, 25 TFs, and a total of 139 edges (Figure 8).

### Verification of hub gene expression

**FIGURE 9:**
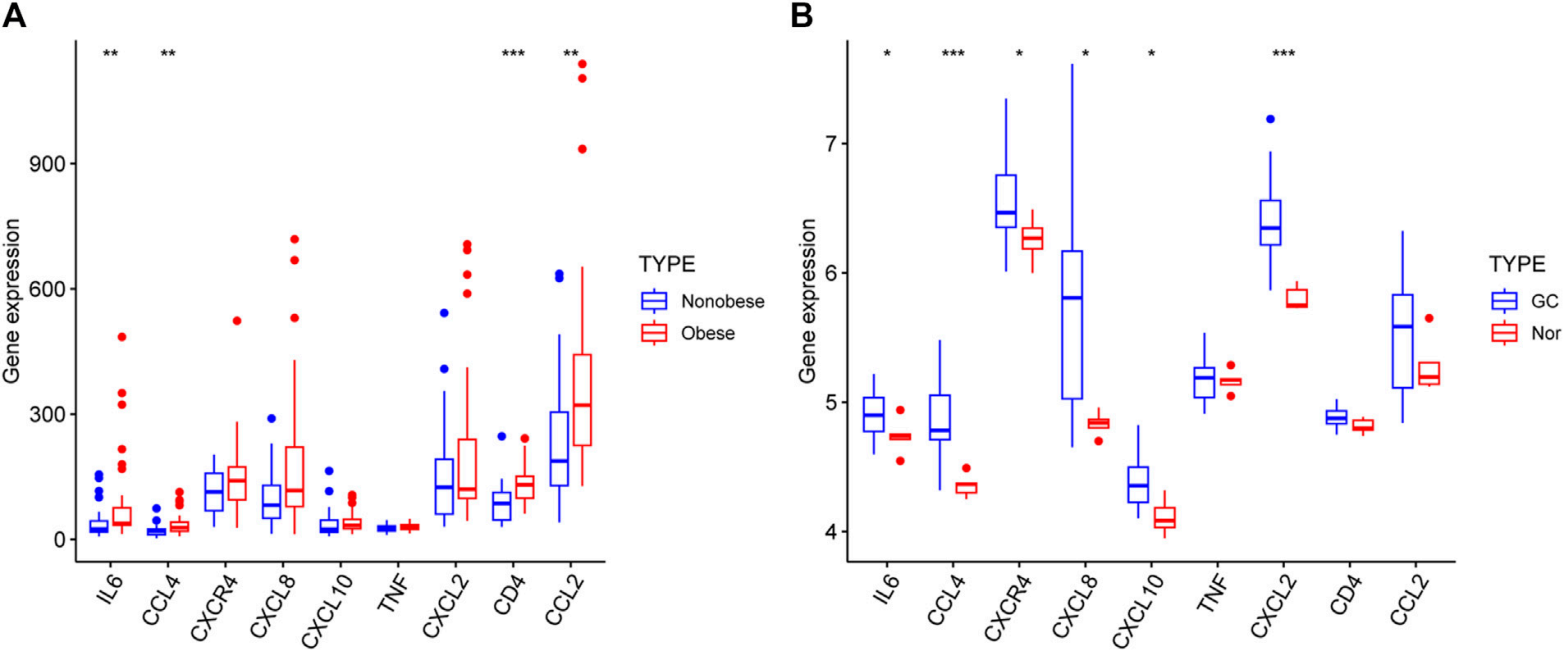
Hub genes expression in the GSE25401 **(A)** and GSE220917 **(B)** datasets. *p < 0.05; **p < 0.01; ***p < 0.001; ****p < 0.0001. GC: gastric cancer, Nor: normal individuals.

The results demonstrated that the expression of nine genes in the two datasets. The levels of *IL6*, *CCL4*, *CD4* and *CCL2* were significantly elevated in the obese group compared with the nonobese group (Figure 9A). In addition, the levels of *IL6*, *CCL4*, *CXCR4*, *CXCL8*, *CXCL10* and *CXCL2* were higher in patients with GC compared with healthy individuals (Figure 9B). However, only *IL6* and *CCL4* were significantly different in both two datasets.

## Discussion

In this research study, we employed bioinformatics techniques to investigate the shared DEGs in patients with obesity and GC. Enrichment analysis showed that these DEGs were mainly enriched in inflammatory and immune-related pathways. PPI network construction, module analysis, GO and KEGG enrichment analyses indicated that these closely connected genes were mainly involved in inflammatory and immune-related pathways. Based on the intersection of the genes obtained by different algorithms of the plugin cytoHubba, we identified nine key genes. Using these nine hub genes, we constructed a regulatory network. The results revealed that *CXCR4*, *CXCL8*, *CXCL10*, *IL6*, *TNF*, *CCL4*, *CXCL2*, and *CCL2* may involve common regulatory factors, such as RELA and NFKB1.

The miRNAs play major roles in cell differentiation and apoptosis, and well as disease progression. Aberrant miRNA expression may promote the development of various cancer types (Xu et al., 2019). Moreover, miRNAs can be used as diagnostic and prognostic markers, and therapeutic targets in cancer (Milanesi et al., 2020). Numerous miRNAs have been implicated in various tumor phenotypes (Garzon et al., 2010). TFs, which are abundant across a vast array of human tissues and cells, perform the critical function of gene expression modulation. They achieve this by identifying and interacting with specific DNA sequences (Lambert et al., 2018). By establishing a gene interaction network, we found that certain TFs (RELA and NFKB1) and miRNAs (has-miR-195-5p and has-miR-106a-5p) may play key roles in the development of obesity and GC.

By validation in the other two datasets, we found that only IL6 and CCL4 of the nine core genes had significant differences in expression in both datasets. They may play a role in the development of obesity and GC.

Virchow proposed that chronic inflammation creates a favorable environment for the development and progression of cancer (Ikwegbue et al., 2019). Some tumors develop from and are closely related to inflammation. Several examples highlight the close association between inflammation and specific types of cancer. For instance, GC is linked to *Helicobacter pylori* infection (Kim and Wang, 2021), nasopharyngeal cancer is associated with herpes virus infection (Okunade, 2020), and liver cancer is associated with hepatitis virus infection (Shen et al., 2023). Chronic inflammation is considered a hallmark of tumorigenesis and progression (Coussens and Werb, 2002). Cytokines produced during chronic inflammation can disrupt normal inflammatory signaling pathways by causing gene mutations, altering the expression and activation of oncogenes, inhibiting apoptosis, and promoting neovascularization (DeNardo et al., 2010). These changes in the cellular environment can create a favorable setting for tumor growth and progression. Moreover, chronic inflammation can contribute to the formation of an immune-suppressive TME. The presence of large numbers of immunosuppressive cells in the TME significantly inhibits the infiltration and function of cytotoxic lymphocytes. These immune cells play a role in suppressing the immune response, thereby promoting tumor initiation and progression (Wen et al., 2022).

Obesity is considered a chronic systemic inflammatory disease (Engin, 2017). It is typically the result of excess nutrients, and inflammation associated with obesity is often found in metabolic tissues, such as white adipose tissue (WAT) (Caputo et al., 2021) WAT consists of different cell types, including adipocytes and immune cells. It produces various pro-inflammatory cytokines and integrates immune signals in a dysfunctional metabolic state. WAT releases inflammatory molecules, such as *IL6*, *TNF*, and *CCL2*, which can contribute to cancer progression by promoting inflammation (Murphy et al., 2018). These cytokines can recruit a variety of immune cells, such as tumor-associated macrophages, which are the most abundant immune cells in the TME. The presence of a large number of inflammatory cells within the TME is associated with cell proliferation, migration, and angiogenesis. Chronic inflammation is also involved in immunosuppression, creating an internal environment conducive to tumorigenesis, infiltration, and metastasis (Zhao et al., 2021). The formation of an inflammatory TME induces the expression of numerous cytokines and inflammatory factors, thereby mediating multiple pathways and promoting tumor growth and invasion (Ben-Baruch, 2020). In summary, the release of pro-inflammatory cytokines from WAT and the recruitment of immune cells contribute to the establishment of a chronic inflammatory environment, which can promote tumor progression.

*IL6* (a cytokine with pleiotropic effects) regulates a wide range of functions related to hematopoiesis, tissue homeostasis, metabolism, and immunity (Jones and Jenkins, 2018). Its dysregulation is implicated in various diseases, such as chronic inflammation, autoimmune disorders, and cancer. *CCL4* (a CC chemokine) induces the recruitment of tumor-associated macrophages and regulatory T cells that exert pro-tumorigenic effects. Other cells in the TME, such as mesenchymal fibroblasts and vascular endothelial cells, are also affected by *CCL4*, contributing to tumor growth (Mukaida et al., 2020). *IL6* and *CCL4* may play important roles in the microenvironment of obesity and GC. Further data analysis is required to comprehensively investigate the importance of these factors.

It is important to acknowledge the limitations of this study. The present analysis was based on microarray data, which have not been experimentally validated. Therefore, further basic research and clinical validation are warranted to investigate the specific signaling pathways of these hub genes, and further clarify the relationship between obesity and GC.

## Conclusion

In this study, we investigated the DEGs in obesity and GC and identified potential common molecular mechanisms underlying the development of both conditions using bioinformatics methods. A PPI network was constructed, and nine hub genes were identified: *CXCR4*, *CXCL8*, *CXCL10*, *IL6*, *TNF*, *CCL4*, *CXCL2*, *CD4*, and *CCL2*. Subsequently, regulatory networks of miRNAs, mRNAs, and TFs were created. Thereafter, the hub genes were validated using different datasets. Finally, *IL6* and *CCL4* were confirmed as the hub genes associated with both obesity and GC. The present findings may facilitate future investigations into underlying mechanisms and predictions of therapeutic targets.

## Data Availability

The datasets presented in this study can be found in online repositories. The names of the repository/repositories and accession number(s) can be found in the article/Supplementary Material.
